# Another Case of Habitual Regurgitation

**Published:** 1874-08

**Authors:** Geo. W. Emery

**Affiliations:** De Pere, Brown Co., Wis.


ARTICLE II.—*Another Case of Habitual Regurgitation, similar to the one reported in former number of* MEDICAL JOURNAL. By GEO. W. EMERY, M.D., De Pere, Brown Co., Wis.

William H——, aged fifty-six, a farmer, consulted me a few months since at my office, then in Paxton, Ill., for what I then discovered to be intermittent fever. In taking the particulars of his case, he informed me that he had a peculiar habit, which he said had existed for twenty-five years, and for which he had consulted several physicians without any beneficial results, and none claiming to have met with a similar case. The following is from my case book:

No cephalalgia nor vertigo; at present has bad taste, and tongue is furred; never subjected to constipation, and appetite has always been good; he regurgitates his food while eating, also several times after meals, and again after repeating the operation of mastication returns food to the stomach, the amount regurgitated decreasing after each time regurgitated; about three hours after meals the eructations cease to be palatable, as they then acquire a bitterish, acid taste, and the patient then expectorates the then much decreased eructations, which appear of a chylous nature.

He also stated, as in Dr. Graham's case, that the pleasure of remastication excelled that of the meal. In this case I found hepatic hypertrophy, with some splenic enlargement, which I think was produced by malarial fever about seven years previous, and which I could not connect in any way with the condition named.

My treatment was not directed to a case of abnormal condition of the stomach, as the patient, as well as myself, considered the case from its long standing beyond any aid. My theory is that it was an acquired habit, and with care in masticating, slowly eating, and opposing the habit, the difficulty could have been removed.

